# Construction of a nomogram to predict weaning-induced pulmonary edema in mechanically ventilated patients with cardiogenic respiratory failure

**DOI:** 10.3389/fmed.2025.1713050

**Published:** 2025-12-03

**Authors:** Fulian Zhang, Li Li, Tingting Zhu, Ke Liu

**Affiliations:** Department of Intensive Care Unit, Chengbei Branch, Hangzhou First People's Hospital, Hangzhou, China

**Keywords:** weaning-induced pulmonary edema, cardiogenic respiratory failure, mechanical ventilation, risk model, APACHE II, RSBI

## Abstract

**Introduction:**

Weaning-induced pulmonary edema (WIPE) is a common but often underrecognized complication in patients undergoing mechanical ventilation for cardiogenic respiratory failure. This study aimed to construct and validate a risk prediction model to predict the occurrence of WIPE in this population.

**Methods:**

A retrospective analysis was conducted on 262 patients with cardiogenic respiratory failure who received invasive mechanical ventilation between April 2021 and December 2023. Clinical characteristics, physiological indicators, and comorbidities were compared to identify independent risk factors for WIPE.

**Results:**

Nine variables were identified as independent risk factors for WIPE: age, smoking history, APACHE II score, NYHA cardiac function grade, RSBI, duration of mechanical ventilation, history of hypertension, left ventricular diastolic dysfunction, and COPD. The constructed logistic regression model demonstrated excellent predictive performance (AUC = 0.884, sensitivity = 0.923, specificity = 0.811). The calibration curve showed good agreement between predicted and observed outcomes (MAE = 0.050).

**Discussion:**

This study developed a preliminary risk prediction model that has the potential to assist clinicians in early identification of patients at high risk of WIPE, but external validation in larger multicenter cohorts is warranted prior to clinical implementation.

## Introduction

Cardiogenic respiratory failure represents a critical clinical syndrome characterized by severe pulmonary congestion, impaired gas exchange, and hypoxemia due to underlying cardiac dysfunction—most commonly left ventricular failure (1). Mechanical ventilation remains the cornerstone of supportive therapy in such patients, as it provides essential oxygenation and reduces the workload of the failing heart and respiratory muscles (2). However, weaning from ventilatory support poses significant challenges, particularly in patients with underlying cardiac conditions (3). Among the various complications associated with ventilator weaning, weaning-induced pulmonary edema (WIPE) is under-recognized but clinically significant. WIPE occurs when the transition from positive-pressure ventilation to spontaneous breathing leads to hemodynamic perturbations that the cardiovascular system cannot accommodate, particularly in patients with impaired diastolic or systolic function (4). The pathophysiological mechanism involves abrupt changes in intrathoracic pressure that increase venous return, left ventricular afterload, and pulmonary capillary hydrostatic pressure, leading to alveolar and interstitial fluid accumulation (5).

The clinical implications of WIPE are considerable. WIPE is a principal contributor to weaning failure, resulting in prolonged mechanical ventilation, increased rates of reintubation, longer ICU stays, higher hospital costs, and greater mortality (6, 7). Accurate and early identification of patients at high risk of WIPE could dramatically improve weaning outcomes and reduce the burden on healthcare systems. The reported incidence of WIPE among patients with cardiogenic respiratory failure or cardiac comorbidities ranges from 10% to over 60%, depending on patient selection and diagnostic modalities (8). However, diagnosing WIPE remains challenging due to the absence of standardized criteria and its overlap with other causes of extubation failure, such as airway obstruction, respiratory muscle fatigue, or residual sedation (9). While tools such as the rapid shallow breathing index (RSBI), minute ventilation, oxygenation index, and spontaneous breathing trial (SBT) tolerance are widely used to evaluate weaning readiness, they are primarily focused on respiratory mechanics and often fail to account for underlying cardiovascular pathology (10). This is especially problematic in patients with heart failure, chronic obstructive pulmonary disease (COPD), or structural cardiac abnormalities, where standard weaning parameters may appear acceptable even when cardiac output cannot sustain spontaneous breathing (11). Several cardiac-specific markers have been proposed to aid in WIPE diagnosis and prediction. For example, studies have shown that elevated extravascular lung water index (EVLWI), increased B-type natriuretic peptide (BNP), hemoconcentration during SBT, and a rise in pulmonary artery occlusion pressure (PAOP) are associated with WIPE (12, 13). However, these methods often require invasive monitoring such as pulmonary artery catheterization or transpulmonary thermodilution, which are not universally available or feasible in many ICUs (14).

Consequently, there is a growing interest in developing non-invasive, clinically accessible, multivariate prediction models that combine both respiratory and cardiovascular parameters. Prior work has identified predictors such as age, APACHE II score, COPD, hypertension, duration of mechanical ventilation, and cardiac dysfunction as key risk factors for weaning failure (15). In particular, the integration of echocardiographic markers of left ventricular diastolic dysfunction (LVDD) has been emphasized as a valuable tool to guide ventilator withdrawal strategies (16). Despite these advancements, few studies have systematically developed and validated a predictive model specifically tailored to WIPE in cardiogenic respiratory failure patients. The existing models are often limited by small sample sizes, narrow inclusion criteria, or a lack of external validation. Moreover, there is a need for models that balance predictive accuracy with ease of clinical implementation—leveraging readily available clinical data without the need for invasive hemodynamic monitoring or specialized biomarkers.

In this context, this study aimed to identify independent predictors of WIPE and to develop a nomogram-based risk prediction model for patients with cardiogenic respiratory failure requiring mechanical ventilation using a cohort of 262 cases. We believe that the implementation of such a model may improve clinical outcomes and enhance the safety and efficiency of ICU care for cardiogenic respiratory failure patients.

## Methods

### Study design and population

This retrospective observational study was conducted in the intensive care unit (ICU) of a tertiary, university-affiliated hospital in China. Data were collected from adult patients admitted between April 2021 and December 2023 who required invasive mechanical ventilation due to cardiogenic respiratory failure. Cardiogenic respiratory failure was defined as hypoxemic respiratory failure secondary to acute left ventricular systolic or diastolic dysfunction, confirmed by echocardiography. Eligible patients had to receive invasive mechanical ventilation for at least 48 h and undergo at least one spontaneous breathing trial (SBT) prior to extubation. Patients were excluded if they had a tracheostomy in place, neuromuscular disorders (e.g., Guillain–Barré syndrome, myasthenia gravis), structural upper airway obstruction, or if they were comatose or hemodynamically unstable during the planned SBT. This study was conducted in accordance with the Helsinki Declaration of 1975 as revised in 2013. This retrospective analysis used de-identified data retrieved from the hospital electronic medical record system, and informed consent for the use of records was obtained from each patient upon admission; therefore, no patient recruitment or follow-up was performed. This study was approved by the Ethics Committee of Hangzhou First People’s Hospital (Approval no. KY-202303023). The reporting of this study adheres to the Strengthening the Reporting of Observational Studies in Epidemiology (STROBE) guidelines (17).

### Group classification

Patients were stratified into two groups: the WIPE group (*n =* 62) and the non-WIPE group (*n =* 200). The diagnosis of weaning-induced pulmonary edema (WIPE) was based on clinical criteria adapted from previous studies (12, 13) and included: (1) development of dyspnea, tachypnea (RR > 30/min), hypoxemia (SpO₂ < 90% on FiO₂ ≥ 40%), or respiratory distress during or within 2 h of SBT or extubation; and (2) evidence of cardiogenic pulmonary edema, defined by any two of the following: new bilateral crackles, radiographic signs of pulmonary congestion, echocardiographic markers of increased left-sided filling pressure, or elevated B-type natriuretic peptide (BNP > 400 pg./mL). Patients who showed stable hemodynamics and respiratory performance without signs of pulmonary edema were assigned to the non-WIPE group.

### Data collection

Clinical data were extracted from the hospital’s electronic medical record system using a structured template. Demographic data included age, sex, and body mass index (BMI). Baseline comorbidities were recorded, including hypertension, chronic obstructive pulmonary disease (COPD), diabetes mellitus, and lifestyle factors such as smoking and alcohol history. The New York Heart Association (NYHA) functional classification was used to grade pre-admission cardiac function. Acute severity at ICU admission was assessed using the Acute Physiology and Chronic Health Evaluation II (APACHE II) score. Respiratory variables were recorded immediately before the SBT and included respiratory rate, tidal volume (VT), minute ventilation (VE), oxygenation index (PaO₂/FiO₂), rapid shallow breathing index (RSBI, calculated as RR/VT in liters), and duration of prior mechanical ventilation (in hours). APACHE II at ICU admission was retained as a composite index of baseline systemic severity and organ-reserve capacity, whereas respiratory parameters were collected immediately before the SBT to reflect acute load tolerance. This temporal integration reflects real-world clinical decision-making, in which both chronic physiologic fragility and current respiratory performance jointly determine weaning outcomes.

Sedative use (Yes/No) and level of consciousness were also noted at the time of SBT. Echocardiography was performed by ICU physicians certified in critical care ultrasonography using standardized protocols. Left ventricular diastolic dysfunction (LVDD) was defined according to the 2009 American Society of Echocardiography and European Association of Echocardiography (ASE/EAE) recommendations, using the average E/e′ ratio, left atrial volume index, and peak tricuspid regurgitation velocity (18).

### Weaning protocol and SBT

Patients were considered for weaning based on readiness criteria including improvement or resolution of the underlying disease, hemodynamic stability, minimal vasopressor support, adequate oxygenation (PaO₂/FiO₂ > 150–200 mmHg on PEEP ≤ 8 cmH₂O), and acceptable mental status. SBT was conducted using T-piece trials or low-level pressure support ventilation (PSV: pressure support ≤ 7 cmH₂O, PEEP = 5 cmH₂O, FiO₂ ≤ 40%) for 30 to 120 min. Vital signs, arterial blood gases, and respiratory effort were monitored throughout. SBT was terminated early in cases of clinical deterioration, and extubation was performed only if the patient passed the SBT based on predefined criteria.

### Statistical analysis

All statistical analyses were performed using SPSS version 26.0 and R version 4.3.0. Continuous variables were tested for normality using the Kolmogorov–Smirnov test. Normally distributed variables were expressed as mean ± standard deviation (x̄ ± s) and compared between the WIPE and non-WIPE groups using independent sample *t*-tests. Non-normally distributed variables were presented as median and interquartile range [M (P25, P75)] and compared using the Mann–Whitney U test. Categorical variables were expressed as frequencies and percentages (n, %) and compared using the chi-square test or Fisher’s exact test as appropriate. To identify risk factors associated with WIPE, univariate logistic regression analysis was performed on all clinical and physiological variables. Variables with a *p* value < 0.05 in univariate analysis were then entered into a multivariate logistic regression model using a forward stepwise likelihood ratio method to determine independent predictors. Odds ratios (ORs) and 95% confidence intervals (CIs) were calculated for all covariates retained in the final model. The prediction model was established based on the multivariate logistic regression results using the “rms” package in R software (version 4.3.0). Calibration of the model was evaluated using a calibration curve and the mean absolute error (MAE) between predicted and actual probabilities of WIPE occurrence. Internal validation of the model was performed using bootstrapping with 1,000 resamples to assess the stability and robustness of model performance indicators. Model fit was evaluated using the Hosmer–Lemeshow goodness-of-fit test and pseudo-R^2^ indices (Cox–Snell and Nagelkerke R^2^). A Hosmer–Lemeshow *p*-value > 0.05 was considered indicative of adequate calibration. Multicollinearity among predictors was assessed by calculating the variance inflation factor (VIF) for each variable, with a VIF < 5 considered indicative of acceptable independence (no multicollinearity). All statistical tests were two-tailed, and a *p*-value < 0.05 was considered statistically significant. Model discrimination was assessed by constructing a receiver operating characteristic (ROC) curve and calculating the area under the curve (AUC). The optimal cutoff value was determined using the Youden index, and corresponding sensitivity and specificity were calculated.

## Results

### Comparison of baseline characteristics

A total of 262 patients with cardiogenic respiratory failure who received invasive mechanical ventilation were included in the final analysis. Of these, 62 patients (23.7%) developed weaning-induced pulmonary edema (WIPE), while 200 patients (76.3%) were successfully weaned without signs of pulmonary edema (non-WIPE group). The comparison of baseline characteristics between the two groups is presented in Table 1.

**Table 1:** Comparison of baseline characteristics between WIPE and non-WIPE groups.

Variable	WIPE group (*n =* 62)	Non-WIPE group (*n =* 200)	t/*χ*^2^/F	*P-*value
Age (years)^a^	76.47 ± 9.87	78.28 ± 9.16	1.48	0.22
Sex (Male/Female)^b^	39 (62.90%) / 23 (37.10%)	119 (59.50%) / 81 (40.50%)	0.25	0.62
BMI (<25 / ≥25 kg/m^2^)^b^	48 (77.42%) / 14 (22.58%)	172 (86.00%) / 28 (14.00%)	2.01	0.16
Smoking history (Yes/No)^b^	33 (53.23%) / 29 (46.77%)	65 (32.50%) / 135 (67.50%)	6.49	**0.011**
Alcohol use (Yes/No)^b^	28 (45.16%) / 34 (54.84%)	79 (39.50%) / 121 (60.50%)	0.28	0.59
APACHE II score^a^	18.71 ± 5.16	16.37 ± 2.86	4.15	**<0.001**
NYHA Cardiac Function (II / III–IV)^b^	29 (46.77%) / 33 (53.23%)	149 (74.50%) / 51 (25.50%)	6.29	**0.012**
Heart rate (beats/min)^a^	102.00 ± 17.14	97.40 ± 13.03	0.58	0.56
Oxygenation index (PaO₂/FiO₂, mmHg)^a^	233.7 ± 27.3	230.5 ± 24.2	1.14	0.26
RSBI (breaths/min/L)^a^	81.97 ± 13.01	73.54 ± 9.08	6.08	**<0.001**
Tidal volume (mL)^a^	411.80 ± 32.66	420.34 ± 34.50	1.34	0.18
Minute ventilation (L/min)^a^	6.02 ± 1.44	6.76 ± 1.57	2.56	**0.011**
Duration of mechanical ventilation (h)^a^	9.78 ± 3.34	9.26 ± 3.25	3.72	**<0.001**
FiO₂ during ventilation (%)^a^	41.28 ± 5.60	42.43 ± 5.71	1.08	0.28
Inspired gas temperature (31 °C / 34 °C / 37 °C)^b^	9 / 9 / 44	21 / 27 / 152	0.17	0.85
Hypertension (Yes/No)^b^	48 (77.42%) / 14 (22.58%)	102 (51.00%) / 98 (49.00%)	5.13	**0.024**
Hyperlipidemia (Yes/No)^b^	10 (16.13%) / 52 (83.87%)	30 (15.00%) / 170 (85.00%)	0.15	0.7
Diabetes mellitus (Yes/No)^b^	17 (27.42%) / 45 (72.58%)	48 (24.00%) / 152 (76.00%)	1.23	0.27
LVDD (Yes/No)^b^	29 (46.77%) / 33 (53.23%)	45 (22.50%) / 155 (77.50%)	7.5	**0.006**
COPD (Yes/No)^b^	33 (53.23%) / 29 (46.77%)	43 (21.50%) / 157 (78.50%)	6.01	**0.014**

^a^Independent-sample *t*-test; ^b^*χ*^2^ test. Continuous variables are expressed as mean ± SD or median (IQR); categorical variables as *n* (%). BMI: Body mass index; APACHE II: acute physiology and chronic health evaluation II; NYHA: The New York Heart Association; RSBI, rapid shallow breathing index; LVDD: left ventricular diastolic dysfunction; COPD: chronic obstructive pulmonary disease.

*p* value smaller than 0.05.

The mean age of the WIPE group was 76.47 ± 9.87 years, slightly younger than the non-WIPE group (78.28 ± 9.16 years), but this difference was not statistically significant (*p* = 0.22). There were no significant differences in sex distribution or body mass index (BMI) between groups. However, smoking history was significantly more prevalent in the WIPE group (53.23%) compared to the non-WIPE group (32.50%) (*p* = 0.011). The WIPE group exhibited significantly higher APACHE II scores (18.71 ± 5.16 vs. 16.37 ± 2.86, *p* < 0.001), indicating more severe baseline illness. Additionally, a higher proportion of WIPE patients had NYHA class III–IV cardiac dysfunction (53.23% vs. 25.50%, *p* = 0.012), suggesting that impaired cardiac reserve may be a key contributor to WIPE. Respiratory indicators also differed notably between groups. Patients in the WIPE group had a significantly higher rapid shallow breathing index (RSBI) (81.97 ± 13.01 vs. 73.54 ± 9.08, *p* < 0.001) and a lower minute ventilation (6.02 ± 1.44 vs. 6.76 ± 1.57 L/min, *p* = 0.011). The WIPE group also experienced a longer mechanical ventilation duration prior to weaning (9.78 ± 3.34 vs. 9.26 ± 3.25 h, *p* < 0.001). No significant differences were found in oxygenation index, tidal volume, or FiO₂ concentration. As for comorbidities, hypertension (77.42% vs. 51.00%, *p* = 0.024), left ventricular diastolic dysfunction (46.77% vs. 22.50%, *p* = 0.006), and chronic obstructive pulmonary disease (COPD) (53.23% vs. 21.50%, *p* = 0.014) were significantly more common in the WIPE group. Other comorbidities, including hyperlipidemia and diabetes mellitus, showed no significant differences (Table 1).

### Variable coding for logistic regression

To construct a predictive model for WIPE, variables were included based on clinical relevance and statistical significance. Continuous variables such as age, APACHE II score, RSBI, minute ventilation, and ventilation duration were entered as original values. Binary categorical variables—including smoking history, NYHA class, hypertension, left ventricular diastolic dysfunction (LVDD), and COPD—were encoded as 0 (no) or 1 (yes). The full coding scheme is shown in Table 2.

**Table 2:** Variable coding for logistic regression.

Variable	Coding
Age	Entered as continuous variable
Smoking history	No = 0, Yes = 1
APACHE II score	Entered as continuous variable
NYHA cardiac function class	Class II = 0, Class III/IV = 1
RSBI (Rapid Shallow Breathing Index)	Entered as continuous variable
Minute ventilation	Entered as continuous variable
Mechanical ventilation duration	Entered as continuous variable
Hypertension	No = 0, Yes = 1
Left ventricular diastolic dysfunction (LVDD)	No = 0, Yes = 1
Chronic obstructive pulmonary disease (COPD)	No = 0, Yes = 1

APACHE II: acute physiology and chronic health evaluation II; NYHA: The New York Heart Association; RSBI, rapid shallow breathing index; LVDD: left ventricular diastolic dysfunction; COPD: chronic obstructive pulmonary disease.

### Multivariate logistic regression analysis

Multivariate logistic regression analysis was conducted to identify independent predictors of WIPE. The results are presented in Table 3. Ten variables were included in the model. Age, smoking history, APACHE II score, NYHA class III–IV, RSBI, duration of mechanical ventilation, hypertension, LVDD, and COPD were found to be statistically significant predictors. Specifically, patients with COPD had an approximately 5.54-fold higher risk of developing WIPE (OR = 5.536, 95% CI: 1.685–18.186, *p* = 0.005), followed by LVDD (OR = 5.151), hypertension (OR = 4.903), and NYHA class III–IV heart failure (OR = 4.043). Higher RSBI, APACHE II score, age, and prolonged ventilation duration also contributed significantly. Minute ventilation was not a statistically significant predictor (*p* = 0.269).

**Table 3:** Univariate and multivariate logistic regression analysis of factors associated with WIPE.

Variable	Univariate analysis			Multivariate analysis			
	OR	95% CI (Lower–Upper)	*P*-value	*β*	SE	Adjusted OR (95% CI)	*P*-value
Age (years)	1.063	1.008–1.124	**0.028**	0.069	0.034	1.072 (1.003–1.145)	**0.041**
Smoking history (Yes vs. No)	3.019	1.318–6.915	**0.009**	1.227	0.575	3.412 (1.106–10.530)	**0.033**
APACHE II score	1.175	1.042–1.323	**0.005**	0.169	0.076	1.184 (1.020–1.374)	**0.026**
NYHA cardiac function (III–IV vs. II)	3.854	1.678–8.851	**0.001**	1.397	0.599	4.043 (1.249–13.092)	**0.02**
RSBI (breaths/min/L)	1.089	1.048–1.132	**<0.001**	0.095	0.024	1.100 (1.050–1.152)	**<0.001**
Minute ventilation (L/min)	0.792	0.572–1.097	0.163	NA	NA	NA	NA
Duration of mechanical ventilation (h)	1.438	1.165–1.775	**0.001**	0.432	0.141	1.540 (1.169–2.030)	**0.002**
Hypertension (Yes vs. No)	4.231	1.652–10.832	**0.003**	1.59	0.578	4.903 (1.578–15.233)	**0.006**
LVDD (Yes vs. No)	4.978	1.881–13.178	**0.001**	1.639	0.598	5.151 (1.594–16.640)	**0.006**
COPD (Yes vs. No)	5.12	1.972–13.300	**0.001**	1.711	0.607	5.536 (1.685–18.186)	**0.005**

Multivariate logistic regression model including variables with p < 0.05 in univariate analysis. APACHE II: acute physiology and chronic health evaluation II; NYHA: The New York Heart Association; RSBI, rapid shallow breathing index; LVDD: left ventricular diastolic dysfunction; COPD: chronic obstructive pulmonary disease.

*p* value smaller than 0.05.

### Nomogram model for predicting WIPE in mechanically ventilated patients with cardiogenic respiratory failure

**Figure 1:**
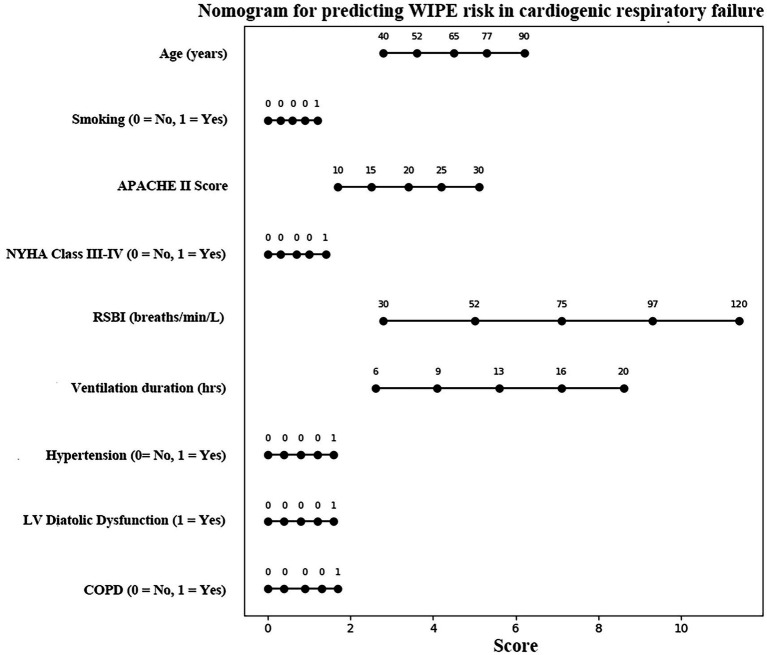
Nomogram model for predicting WIPE in mechanically ventilated patients with cardiogenic respiratory failure. For instance, a 70-year-old smoker (20 pack-years) with APACHE II = 20, NYHA III, RSBI = 85 breaths/min/L, hypertension, COPD, and echocardiographic LV diastolic dysfunction would yield a total of ~240 points on the nomogram, corresponding to a predicted WIPE probability ≈ 0.82. Such a patient would benefit from pre-emptive non-invasive ventilation and diuretic optimization prior to extubation.

To facilitate individualized risk prediction for weaning-induced pulmonary edema (WIPE) in patients undergoing mechanical ventilation due to cardiogenic respiratory failure, we developed a nomogram model based on a multivariate logistic regression analysis. This model integrates key clinical and physiological variables to allow intuitive, bedside risk estimation. Nine variables were identified as independent predictors of WIPE and included in the nomogram: age, smoking history, APACHE II score, NYHA cardiac function class, rapid shallow breathing index (RSBI), mechanical ventilation duration, hypertension, left ventricular diastolic dysfunction (LVDD), and chronic obstructive pulmonary disease (COPD). The logistic regression model was expressed as: Z = 0.069 × Age + 1.227 × Smoking + 0.169 × APACHE II Score + 1.397 × NYHA Class (III–IV) + 0.095 × RSBI + 0.432 × Ventilation Duration + 1.590 × Hypertension + 1.639 × LVDD + 1.711 × COPD − 25.366. The predicted probability of WIPE is calculated by: *p* = 1 / (1 + e^-Z^). Based on the above equation, a nomogram was constructed to visually depict the contribution of each variable to WIPE risk (Figure 1). Each horizontal axis corresponds to a predictor, and its associated scale quantifies the relative contribution to the total risk score. To use the nomogram: Locate the patient’s value for each predictor on its respective axis, draw a vertical line upward to determine the corresponding point value, sum all individual points to obtain a total score, and match the total score to the bottom risk scale to determine the predicted probability of WIPE. This nomogram transforms a statistical regression model into an accessible graphical interface, allowing ICU physicians to rapidly assess WIPE risk before extubation. High-risk patients—such as those with elevated RSBI, reduced cardiac function, or COPD—can be identified in advance and benefit from targeted interventions such as delayed weaning, optimized cardiac therapy, or prophylactic noninvasive ventilation.

### Prediction model performance validation

**Figure 2:**
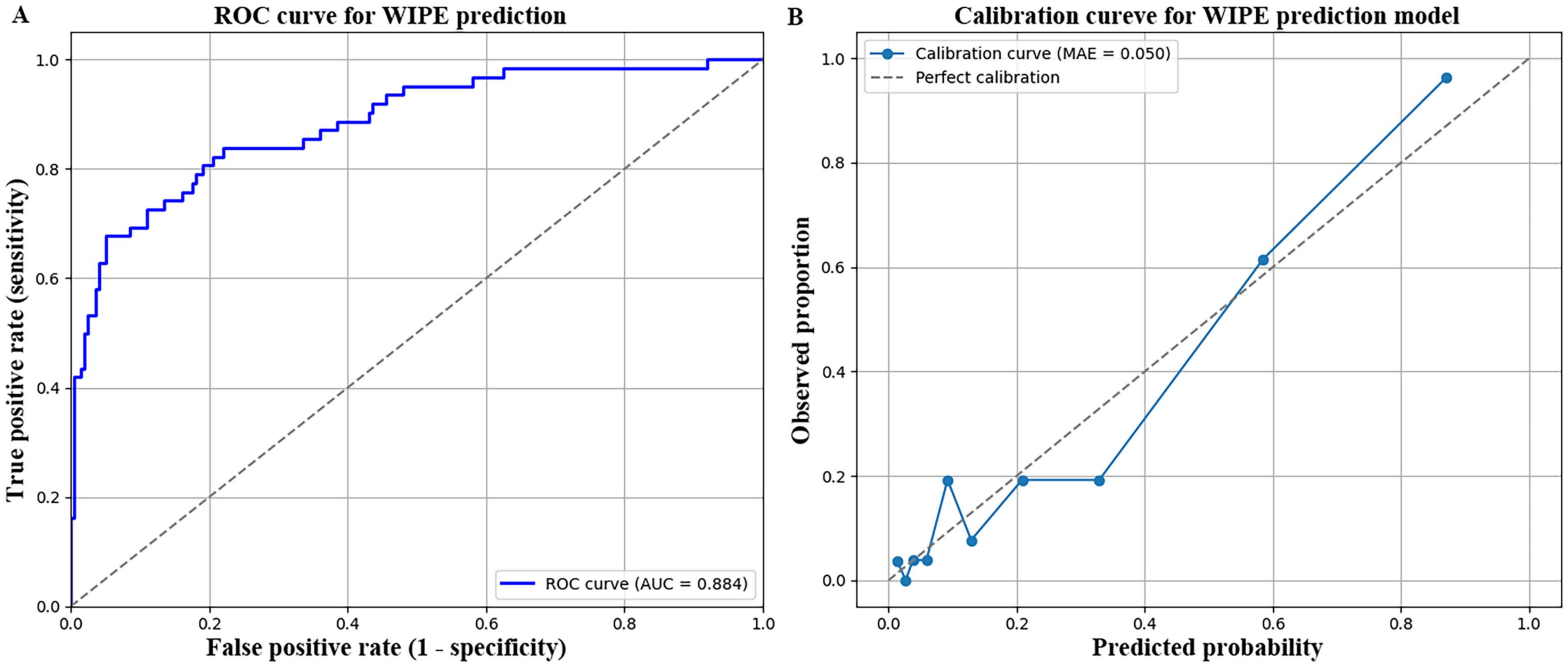
Prediction model performance validation. **(A)** ROC curve for model predication. **(B)** Calibration curve for model prediction.

The predictive model exhibited excellent discriminatory power in identifying patients at risk of weaning-induced pulmonary edema (WIPE). Based on the full dataset (*n =* 262), the area under the receiver operating characteristic (ROC) curve (AUC) was 0.884, indicating a high degree of accuracy in distinguishing between WIPE and non-WIPE cases (Figure 2A). At the optimal cutoff point (determined by the Youden index), the model achieved a sensitivity of 0.923 and a specificity of 0.811. To assess calibration, the model’s predicted probabilities were compared to the observed outcomes. The mean absolute error (MAE) between predicted and actual probabilities was 0.050 (Figure 2B), suggesting excellent agreement and calibration of the model across the range of predicted risks. After internal validation with 1,000 bootstrap resamples, the optimism-corrected AUC was 0.872 and the calibration slope was 0.98, indicating only slight optimism and good internal stability of the model. The model demonstrated good overall fit, with a Hosmer–Lemeshow χ^2^ = 6.74, *p* = 0.56, confirming no significant deviation between predicted and observed outcomes. The Nagelkerke R^2^ was 0.42 and Cox–Snell R^2^ was 0.31, indicating that approximately 31–42% of the variation in WIPE occurrence was explained by the model. Multicollinearity diagnostics showed all variance inflation factors (VIF) < 2.0, suggesting that predictors were statistically independent and did not inflate standard errors.

## Discussion

The current study developed and validated a multivariate logistic regression model to predict WIPE in patients undergoing invasive mechanical ventilation due to cardiogenic respiratory failure. Based on clinical data from 262 patients, the model identified nine independent risk factors and demonstrated strong predictive performance and calibration. These findings suggest that a structured, data-driven approach can significantly enhance clinical decision-making during ventilator weaning in cardiac patients.

WIPE represents a subtype of weaning failure caused by hemodynamic decompensation during or after spontaneous breathing trials (SBTs), often due to abrupt shifts in intrathoracic pressure and volume status, which overwhelm compromised cardiac function (1). Several studies have highlighted underlying cardiac pathology, particularly LVDD, as central to the pathophysiology of WIPE (12, 19). Our study confirms this association: LVDD was independently associated with a more than five-fold increased risk of WIPE, echoing findings by Almeida et al. (20), who established LVDD as a potent predictor of weaning failure in mechanically ventilated patients. Beyond cardiac mechanics, respiratory indices also played a pivotal role. The rapid shallow breathing index (RSBI), a composite index of respiratory frequency and tidal volume, was significantly elevated in the WIPE group. This aligns with previous evidence indicating RSBI’s strong sensitivity to ventilatory inefficiency and respiratory muscle fatigue (3, 7). Furthermore, Saiphoklang et al. (10) demonstrated that RSBI correlated with handgrip strength, underscoring its association with overall muscular function and weaning tolerance. The APACHE II score, another robust predictor in our model, reflects overall illness severity and was significantly higher in WIPE patients. Similar findings have been reported by Wang et al. (21), who showed that APACHE II-based models reliably stratify extubation risk in ICU populations. In combination with mechanical ventilation duration—another significant predictor—this suggests that systemic burden and prolonged respiratory support may synergistically compromise cardiopulmonary stability (22).

Importantly, hypertension and COPD emerged as prominent risk factors in our study. Hypertension, which often leads to concentric left ventricular hypertrophy and impaired compliance, predisposes patients to elevated filling pressures and pulmonary edema during the stress of weaning (23). Similarly, COPD contributes to dynamic hyperinflation, increased right ventricular afterload, and decreased cardiac preload, all of which can provoke or exacerbate WIPE (8, 24). The interaction between these chronic diseases and acute respiratory support highlights the multifactorial nature of extubation failure in cardiac populations. Smoking history was another significant factor, reinforcing the contribution of cumulative pulmonary injury and systemic inflammation in weaning intolerance (25). Moreover, smoking is frequently comorbid with both COPD and cardiovascular disease, potentially amplifying overall risk. Previous epidemiological studies from Chinese and international cohorts have identified smoking as a key determinant in both respiratory decompensation and post-extubation failure (25).

The model’s performance metrics further validate its clinical utility: an AUC of 0.884 indicates good discrimination. These metrics surpass those of prior WIPE-related models, many of which relied on limited variables or subjective assessments. The ROC curve showed excellent sensitivity (0.923) and specificity (0.811) at the optimal cutoff, confirming the model’s potential for real-time clinical decision support. The nomogram based on regression coefficients improves bedside usability. Clinicians can input readily available variables (e.g., age, RSBI, APACHE II score, presence of comorbidities) to derive an individualized WIPE risk estimate. Nomograms have been successfully integrated into critical care settings to guide interventions such as prophylactic noninvasive ventilation (NIV) or pre-weaning hemodynamic optimization (26, 27). Although the present model included nine predictors (EPV ≈ 6.9), which is lower than the ideal 10–20 threshold, bootstrap internal validation with 1,000 resamples demonstrated good discrimination (AUC = 0.872) and calibration (slope = 0.98). These results suggest limited overfitting. Nevertheless, the relatively small event number may affect coefficient stability, and future multicenter validation with penalized regression will be required to confirm model robustness. Our study also aligns with recent findings that emphasize the utility of biomarkers and echocardiographic measurements in predicting weaning outcomes. Dres et al. (28) reported that elevated B-type natriuretic peptide (BNP) levels and extravascular lung water (EVLW) were strongly associated with WIPE, supporting the idea that subclinical fluid overload and cardiac strain may precede clinical decompensation. Incorporating such objective parameters into future iterations of the model may further enhance its performance.

Several limitations should be acknowledged. First, this was a single-center retrospective study, which may limit generalizability. Second, the diagnosis of WIPE relied on clinical and imaging findings rather than invasive hemodynamic measurements (e.g., pulmonary artery occlusion pressure > 18 mmHg), which could introduce some misclassification bias. However, our non-invasive composite diagnostic approach was based on validated frameworks such as those proposed by Dres et al. (12) and Lamia et al. (13), which demonstrated a strong correlation between B-type natriuretic peptide (BNP), echocardiographic E/e′ ratio, and invasively measured filling pressures (12, 13). In contemporary ICU practice, invasive catheterization is often impractical; thus, pragmatic surrogates such as BNP > 400 pg./mL, bilateral crackles, and echocardiographic evidence of elevated left ventricular filling pressure represent accepted diagnostic standards. Although invasive confirmation was not performed, this composite method aligns with prior prospective validation studies and mirrors real-world ICU practice, supporting clinical applicability despite potential diagnostic uncertainty. Third, while bootstrap resampling provided internal calibration, absence of split-sample or external validation may limit model reproducibility. We are initiating a multicenter cohort study across three Zhejiang ICUs to externally validate the model and evaluate temporal stability. Future work will also test recalibration using penalized regression or Bayesian shrinkage to ensure portability. Additionally, regional differences in smoking behavior and COPD prevalence may influence the model’s external generalizability. Epidemiological studies have shown that smoking rates, air pollution levels, and COPD phenotypes vary across China, with northern regions exhibiting higher tobacco exposure and particulate pollution, while southern regions have a greater contribution from biomass fuel exposure. These variations may alter cardiopulmonary vulnerability and modify the association between baseline comorbidities and WIPE risk. Therefore, future multicenter validation should include diverse regional cohorts and, if necessary, apply region-weighted coefficients or mixed-effects logistic modeling to enhance model adaptability and generalizability.

In conclusion, the current study developed a preliminary nomogram incorporating respiratory, hemodynamic, and comorbidity-related variables to estimate the risk of weaning-induced pulmonary edema in patients with cardiogenic respiratory failure. While the model demonstrated good discrimination and calibration in internal validation, its retrospective single-center design and modest event number warrant cautious interpretation. The nomogram should therefore be viewed as an exploratory decision-support tool rather than a ready-to-implement clinical instrument. Future multicenter cohorts with larger sample sizes and prospective external validation are essential to confirm generalizability, refine model coefficients, and evaluate its practical impact on weaning strategies and patient outcomes.

## Data Availability

The raw data supporting the conclusions of this article will be made available by the authors, without undue reservation.
